# Dealing with uncertainty

**DOI:** 10.3205/zma001668

**Published:** 2024-02-15

**Authors:** Sigrid Harendza

**Affiliations:** 1 Universitätsklinikum Hamburg-Eppendorf, III. Medizinische Klinik, Hamburg, Germany

## Editorial

Why does this man have shortness of breath? Is it a heart attack or a pulmonary embolism? Or is it more likely to be severe pneumonia or even corona? Does he actually smoke? Or is he perhaps immunocompromised? Which test is best? Lab results first or a quick X-ray? And should he be given a blood thinner to be on the safe side in case it is a heart attack? – This sounds like a tutorial for problem-based learning in medical school, but in reality it is normal everyday medical practice in surgeries and hospitals – and it is filled with uncertainty that doctors have to deal with. So far, medical students learn little about how to deal with such uncertainty. On the contrary: the widespread learning and testing with multiple-choice questions suggests the certainty that there is always a correct answer. If a feeling of uncertainty arises, for example because the next diagnostic steps are not clear, this can trigger stress in medical students [1]. However, in a learning environment where students constantly have to prove their knowledge and good grades, uncertainty is often suppressed [2]. Students even develop strategies to hide their uncertainty [3]. In order to enable professional medical behaviour in the sense of the role model of the National Competence-Based Learning Objectives Catalog Version 2.0 [https://nklm.de/zend/objective/list/orderBy/@objectivePosition/modul/200553], students should also learn how to deal professionally with uncertainty. 

Uncertainty occurs in two forms: as aleatoric uncertainty, caused by the unpredictable randomness of events, and as epistemic uncertainty, which is due to a lack of knowledge [4]. The latter becomes apparent when discussing patients in the context of clinical decision-making, when the realization sets in that one’s own understanding is not complete or that contradictory findings are available. Instead of using this feeling of uncertainty, which arises from the fact that one’s own knowledge has reached its limit, as a starting point for gaining further knowledge, medicine tends to try to counter this form of uncertainty with algorithms and checklists [5]. This leads medical students – and doctors – to believe that patients’ problems can be solved by working through algorythms and checklists in a structured way. Although this often does not lead to better solutions, it does create less uncertainty. A “tolerance of ambiguity” scale [6] or an “intolerance of uncertainty” test [7] can be used to determine the degree to which people consider uncertainty to be a challenge or a threat [5]. This difference has a decisive influence on how well people solve problems. Situational contexts also play a role. Uncertainty is less tolerated under time pressure because people want to find a solution quickly and are then more likely to make mistakes [5]. Under stress, even experts tend to use a method they are familiar with to solve a problem and not think about whether there could be a more efficient or faster method [5]. Treatment errors can be the price for such behaviour.

In order to learn how to deal with uncertainty, which is inextricably linked to medical work, we need a learning culture in medical studies that makes it possible to ask questions, discuss points of view in a reasoned manner, admit mistakes, and reflect on one's own uncertainty. It was shown that a change in the tolerance of ambiguity in medical students was significantly positively associated with a change in empathy over the course of their studies [8]. In this issue, Schrötter et al. report on the identification of different empathy profiles in medical students in a cross-sectional study [9]. They found that 9th semester students showed a tendency towards unreflected, stressful empathy and only in a third of cases appeared to be able to regulate their own emotions appropriately in order to protect themselves from emotional overload. In this issue, Hajduk et al. show a cross-sectional study in which 12.4% of the participating students stated that they used psychoactive substances to improve performance or for emotional regulation, particularly during exam preparation [10]. In another study in this issue, Hahn et al. found that gratitude has a very small influence on the resilience of medical students, but that optimism as a mediating factor significantly strengthens the resilience of medical students [11].

These studies show interesting results in order to better understand the learning culture and thus also offer starting points for further investigations into how professional behaviour and, in particular, dealing with uncertainty could be strengthened. The study by Drossard and Härtl on digital peer mentoring in this issue also provides an interesting approach in this regard. The authors were able to show that medical students felt less anxious in the first semester as a result of peer mentoring, that they were able to organize themselves better and that peer mentoring had positive effects on both the peer mentees and the peer mentors [12]. The fact that interprofessional small group teaching also contributes to the learning culture in addition to content-related learning effects is already shown in the study by Schneider et al. in this issue by the title: “Great, what we have learned from each other!” [13].

## Author’s ORCID

Sigrid Harendza: 0000-0002-7920-8431

## Competing interests

The author declares that she has no competing interests.

## References

[1] Lally J, Cantillon P (2014). Uncertainty and ambiguity and their association with psychological distress in medical students. Acad Psychiatry.

[2] Kennedy TJT, Regeher G, Baker GR, Lingard L (2008). Point-of-care assessment of medical trainee competence for independent clinical work. Acad Med.

[3] Lingard L, Garwood K, Schryer CF, Spafford MM (2003). A certain art of uncertainty: case presentation and the development of professional identity. Soc Sci Med.

[4] Kiureghian AD, Ditlevsen O (2009). Aleatory or epistemic? Does it matter?. Struct Saf.

[5] Jackson M (2023). Uncertain: the wisdom and wonder of being unsure.

[6] Lietz A (2023). Measuring Tolerance for Ambiguity: A German-language adaption and validation of the tolerance for ambiguity scale (TAS). Meas Instrum Soc Sci.

[7] Carleton RN, Norton MAPJ, Asmundson GJG (2007). Fearing the unknown: A short version of the Intolerance of Uncertainty Scale. J Anxiety Disord.

[8] Geller G, Grbic D, Andolsek KM, Caulfield M, Roskovensky L (2021). Tolerance for ambiguity among medical students: patterns of change during medical school and their implications for professional development. Acad Med.

[9] Schrötter S, Müller B, Kropp P (2024). Comparison of empathy profiles of medical students at the start and in the advanced clinical phase of their training. GMS J Med Educ.

[10] Hajduk M, Tiedemann E, Romanos M, Simmenroth A (2024). Neuroenhancement and mental health in students from four faculties. A cross-sectional questionnaire study. GMS J Med Educ.

[11] Hahn N, Brzoska P, Kiessling C (2024). On the correlation between gratitude and resilience in medical students. GMS J Med Educ.

[12] Drossard S, Härtl A (2024). Development and implementation of digital peer mentoring in small groups for first-year medical students. GMS J Med Educ.

[13] Schneider S, Anders P, Rotthoff T (2024). “Great, what we have learned from each other!” – Bedside teaching in interprofessional small groups using the example of Parksinon’s diseas. GMS J Med Educ.

